# Recellularized lymph node scaffolds with human adipose-derived stem cells enhance lymph node regeneration to improve lymphedema

**DOI:** 10.1038/s41598-023-32473-z

**Published:** 2023-04-03

**Authors:** Hyo Jin Kang, Soo Young Moon, Baek-Kyu Kim, Yujin Myung, Ju-Hee Lee, Jae Hoon Jeong

**Affiliations:** 1 Department of Biomedical Laboratory Science, Honam University, Gwangju, 62399 Republic of Korea; 2 Department of Plastic and Reconstructive Surgery, Seoul National University Bundang Hospital, 82, Gumi-ro 173 Beon-gil, Bundang-gu, Seongnam-si, 13620 Gyeonggi-do Republic of Korea; 3 College of Korean Medicine, Dongguk University, Goyang, 10326 Gyeonggi-do Republic of Korea

**Keywords:** Stem cells, Mesenchymal stem cells, Regeneration, Biomaterials

## Abstract

To overcome the limitations of lymphedema treatment, human adipose-derived stem cells (hADSCs) were injected into decellularized lymph nodes to produce a recellularized lymph node-scaffold, and the effect of lymphangiogenesis was investigated in lymphedema animal models. Axillary lymph nodes were harvested from Sprague Dawley rats (7 weeks old, 220–250 g) for decellularization. The decellularized lymph nodes were performed and PKH26-labeled hADSCs (1 × 10^6^/50 µL) were injected in the decellularized lymph node-scaffolds. Forty rats were equally divided into four groups: lymphedema as control group, hADSC group, decellularized lymph node-scaffold group, and recellularized lymph node-scaffold group. The lymphedema model was made by removing inguinal lymph nodes, and hADSCs or scaffolds were transplanted. Histopathological assessments were performed by hematoxylin and eosin and Masson’s trichrome staining. Lymphangiogenesis was evaluated by Immunofluorescence staining and western blot. Decellularized lymph nodes showed virtually complete absence of cellular material and maintenance of lymph node architecture. The hADSCs were significantly observed in recellularized lymph node-scaffolds group. The recellularized lymph node-scaffold group was histologically similar to normal lymph nodes. The vascular endothelial growth factor A and lymphatic vessel endothelial hyaluronan receptor 1 (LYVE-1) in immunofluorescence staining were highly expressed in recellularized lymph node-scaffolds group. Also, the expression of LYVE-1 protein significantly increased in recellularized lymph node-scaffold group compared with others. Recellularized lymph node -scaffold had a much better therapeutic effect than stem cells or decellularized lymph node-scaffold alone, and induced stable lymphangiogenesis.

## Introduction

Lymphedema is a chronic disease in which the accumulation of lymphatic fluid increases, causing swelling that can cause changes in the skin and tissues. The continuous accumulation of protein-rich fluids exceeds the capacity of the lymphatic system, resulting in edema including the arms, legs, genitals, face and neck^1^. The patient could experience heaviness and fullness. These symptoms develop gradually over time, the discomfort associated with lymphedema can interfere with daily activities. When lymphedema in the upper and lower extremities progresses, pain intensifies and movement is restricted. Moreover, patients with lymphedema are at increased risk of infection in the area. Lymphedema is on the rise for a variety of causes and affects millions of people throughout the world. Primary or congenital lymphedema is rare, but usually results from mutations in the vascular endothelial growth factor receptor 3 (VEGFR3) associated with lymphangiogenesis^2^. Secondary lymphedema is caused by body flow disorders such as infection, inflammation, and injury, and lymphadenectomy and radiation therapy for malignant tumors are known to be the major causes^2,3^. In particular, the risk of developing lymphedema after breast cancer treatment has been reported to be approximately 15–20%, and the exact number of patients suffering from lymphedema is between 140 million and up to 300 million worldwide^4^. Representative treatment of lymphedema is a physical treatment included massages, lymphatic drainages, the application of different kind of compression garments, and intermittent pneumatic compression^5^. Various surgical methods such as lymph node transfer and lymphovenous anastomosis have been tried. However, these surgical methods also have complications and limitations^4^. Recently, regenerative medicine studies using cells, growth factors, scaffolds and mechanical stimulation have been shown to further improve their functionality^6^. Especially, the studies about lymph node regeneration using human adipose-derived stem cells (hADSCs) have been performed, and it has been demonstrated that they differentiate into lymphatic endothelial cells (LECs) and secrete lymphangiogenic growth factors^7^.

Decellularization of tissues or organs is used in regenerative medicine by removing cellular material to reduce immunological reactivity and manufacturing a biocompatible scaffold that preserves the three-dimensional structure and biochemical content of the original tissue^8^. Decellularized allograft or xenograft tissue has gained considerable favor for developing three-dimensional biological scaffolds to restore complex organ function. Decellularization of tissues is to develop a scaffold with low antigenicity by removing the cellular materials and nucleic acids^9^. The usefulness of decellularized scaffolds is widely concentrated in tendons, bladder, blood vessels, kidneys and lungs, but few in lymph nodes^10^. Cuzzone et al. succeeded in decellularization of lymph nodes for the first time and presented the possibility of maintaining the structure of the extracellular matrix (ECM) and delivering cells in vivo^9^.

In addition, cell therapy for treatment of lymphedema is being actively conducted. Stem cells have various effects such as anti-inflammatory, anti-fibrotic, and tissue regeneration promotion, so they can promote lymphatic regeneration and improve interstitial fluid drainage^11^. Cell therapy included stem cells such as d bone marrow-derived mesenchymal stem cells (BM-MSCs), ADSCs, human LECs, and regulatory T cells, and studies including growth factors such as VEGF-C were conducted^12–15^. However, low cell survival and engraftment due to immune response and cell loss are major limitations of cell therapy.

We developed a decellularized lymph node-scaffold for the treatment of lymphedema. In addition, to overcome the limitations of cell therapy, the decellularized lymph node-scaffolds were recellularized by hADSCs, and the effect of lymphangiogenesis was investigated in lymphedema animal models.

## Results

### Decellularization and recellularization of lymph node scaffolds

**Figure 1:**
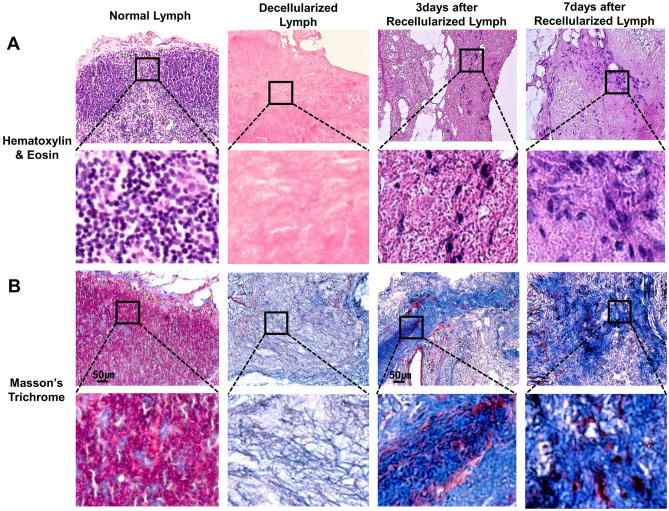
Histopathological assessment of lymph node decellularization and recellularization. (**A**) Hematoxylin &eosin staining of lymph node scaffolds; native lymph node, decellularization treated with 0.075% sodium dodecyl sulfate, recellularization with ADSCs (1 × 10^6^/50 µL). (**B**) Masson’s trichrome staining showing muscle (red) and collagen (blue) deposition.

To identify the decellularized and recellularized lymph node-scaffolds, the tissues were stained by hematoxylin & eosin and Masson’s trichrome. In hematoxylin & eosin staining, normal lymph nodes have a capsule, cortex, and medulla and are filled with lymphatic tissue components such as macrophages, lymphocytes-T cell, and -B cells. Contrary, the decellularized lymph nodes showed virtually complete absence of cellular material and maintenance of lymph node architecture compared to normal lymph nodes. On day 3 of hADSCs recellularization, hADSCs were partially observed in the lymph node-scaffolds, and more hADSCs were observed at day 7 (Fig. 1A).

In Masson’s trichrome, the capsule of the normal lymph node is composed of collagen and bounded, and a collagen scaffold is formed as a whole. After decellularization, the scaffold structure is mostly composed of collagen and some muscle is distributed between the collagen structures. On day 3 of hADSCs recellularization, the distribution of collagen in the scaffold increased, and on the day 7, the collagen distribution was abundant and the structure became more robust (Fig. 1B).

### hADSCs tracing after injection and recellularization in lymphedema

To confirm the effect of recellularization, hADSCs (1 × 10^6^ cells/50 µL) were labeled with PKH26 (red fluorescence) and injected directly into lymphedema animals or transplanted with recellularized lymph node scaffolds. The lymphedema rats were sacrificed on day 7 and 14 for PKH26-labeled hADSC tracking.

**Figure 2:**
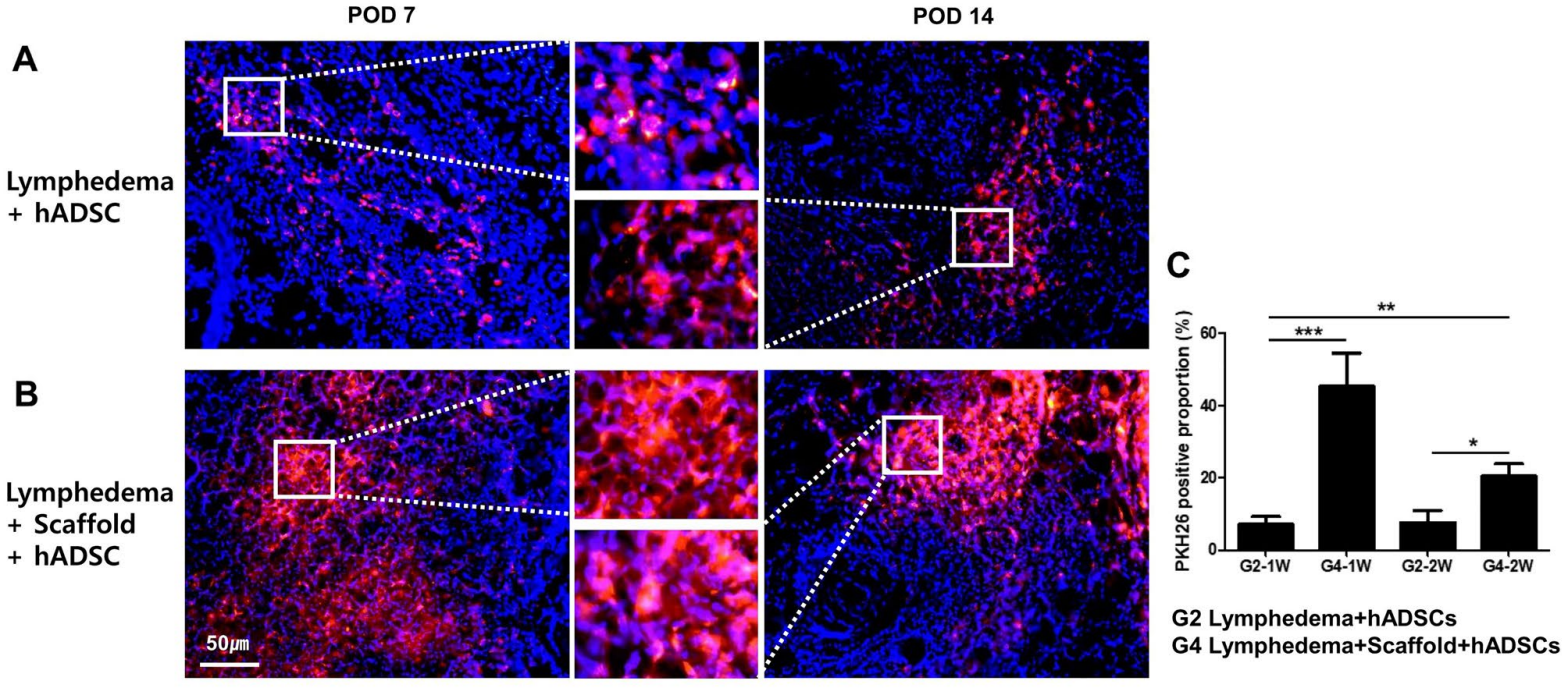
Fluorescent dye-labeled human adipose-derived stem cell (hADSC) tracing. (**A**) PKH26-labeled hADSCs (1 × 10^6^/50 µL) were directly injected into the inguinal lymph nodes site of lymphedema models. (**B**) The lymph node scaffolds were recellularized by PKH26-labeled hADSCs and were transplanted. Large numbers of PKH26-labeled ADSCs were detected in lymph node scaffolds from postoperative day 7 and 14. (**C**) Bar graphs showing that rat in the recellularized LN-scaffold group had the greatest number of PKH26-positive cells (*P < 0.05, ** *P* < 0.01, ***P < 0.001).

When hADSCs were directly injected, cells were observed in the scaffold on postoperative day 7, and PKH26-labeled hADSCs were still identified on postoperative day 14 (Fig. 2A). The PKH25-labeled hADSCs were measured manually in five microscopic fields in each stained sample. At 1 week, PKH25-labeled hADSCs increased about six folds in the recellularized lymph node-scaffold group compared with ADSCs injection group (G2-1W, 7.12 ± 2.18% vs. G4-1W, 45.41 ± 9.11%, *P* < 0.001, Fig. 2A,C), and about three folds at 2 weeks (G2-2W, 7.62 ± 3.33% vs. G4-2W, 20.43 ± 3.43%, *P* < 0.05, Fig. 2B,C). Therefore, the effect of ADSC treatment can be maximized by recellularizing the lymph node scaffold rather than directly injecting the ADSCs, and it was confirmed that hADSCs were continuously maintained over time.

### Histopathological assessment

**Figure 3:**
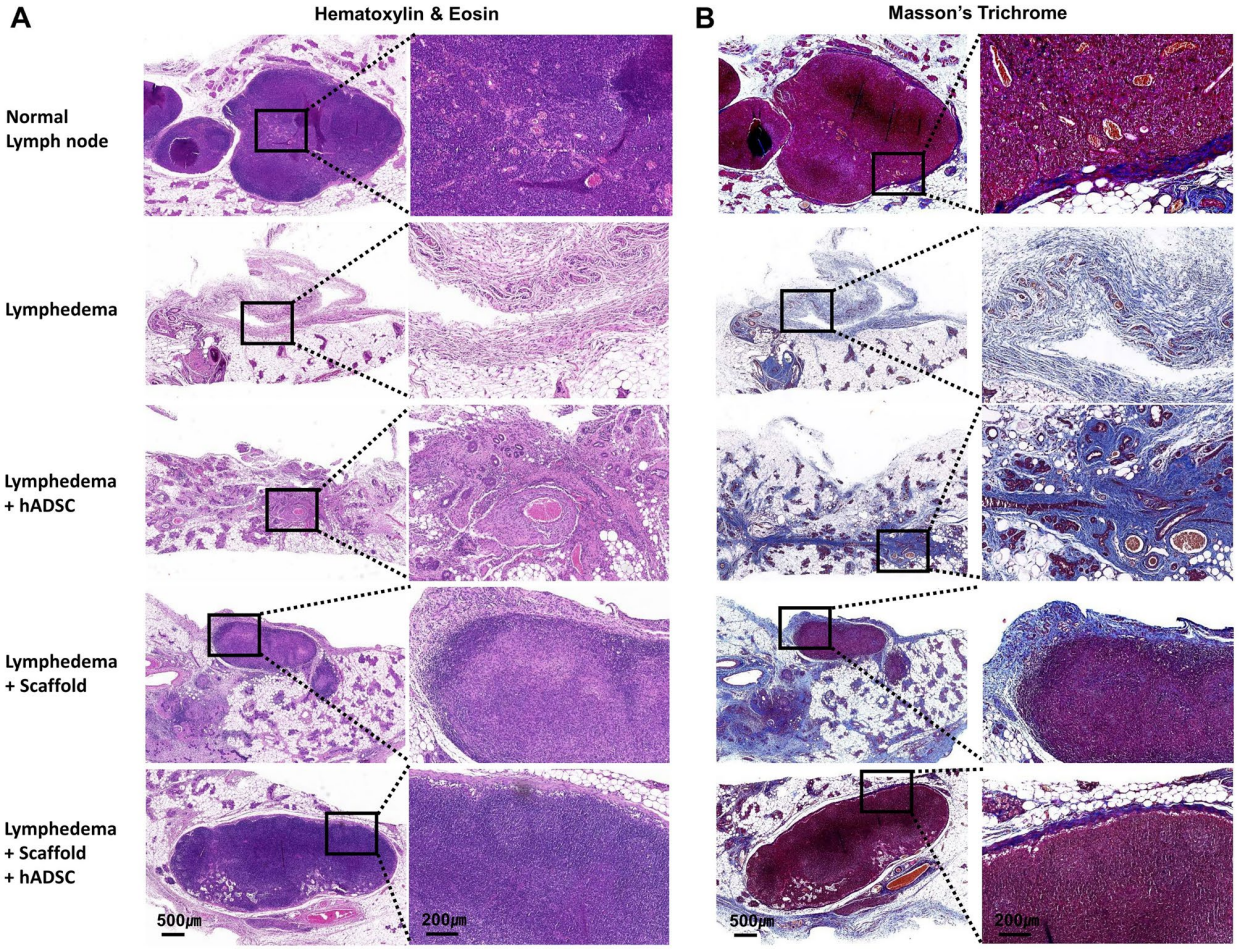
Histopathological observation of recellularizied lymph node scaffold transplantation. (**A**) Hematoxylin and eosin staining of representative inguinal lymph nodes was performed at 14 days postoperatively. No lymph nodes were observed in lymphedema and ADSCs treatment groups, and lymph node scaffolds were observed in scaffold and recellularized lymph node-scaffold groups. (**B**) The inguinal lymph nodes slide sections were stained with Masson's trichrome; collagen stained blue, muscle stained red, and nuclei stained black. In lymphedema and ADSCs treatment groups, the proportion of collagen was high, but in ADSCs treatment group, some muscle were observed around the ADSCs transplantation. In scaffold and recellularized lymph node-scaffold groups, a collagen layer was observed surrounding the lymph node scaffold, and a large number of cell nuclei were observed in the lymph node. Scale bars: 500 µm, and 200 µm.

Inguinal lymph node tissues were stained with hematoxylin and eosin and masson's trichrome on postoperative day 14 for histological observation (Fig. 3). In the lymphedema group, the removed inguinal lymph node remained empty and no signs of tissue regeneration were observed. Plexus, femoral artery, and vein were observed around the removed lymph nodes. Large cells such as hADSCs and a large number of lymphocytes of small size were observed in hADSCs group. Lymphocytes are presumed to be released or migrated by removal of lymph nodes. Although regeneration of lymph nodes was not observed, circular structures such as blood vessels exist. After decellularized lymph node-scaffold transplantation, it was located in the inguinal lymph node and had a lymph node-like shape, but the capsule boundary was not clear, and it seemed that the lymph node was being reconstructed because it was markedly different from the normal lymph node. Lymphocytes and fibroblasts were observed in the form of agglomerates. The recellularized lymph node-scaffold group was histologically similar to normal lymph node, and the capsule of lymph node was clear. Also, cortex and nodules were observed. Many blood vessels exist around the transplanted recellularized lymph node-scaffold, and some myocytes, which are considered to have differentiated hADSCs, were also observed inside the lymph nodes.

In masson's trichrome staining, a capsule form composed of collagen was observed where the inguinal lymph node was removed, and almost no collagen or cellular components were observed around it. Collagen deposition, some cells, and blood vessels were observed around the hADSCs injection site, and traces of lymph node capsules were confirmed.

The transplanted decellularized lymph node-scaffold had a collagen layer similar to a capsule of lymph node, and was significantly thicker than the normal lymph node capsule, and in some cases the boundaries were not clear. In the recellularized lymph node-scaffold with hADSCs group, there is almost no collagen disposition, and a thin band-shaped collagen capsule surrounds the lymph node and clearly demarcates it. Scale bars: 500 µm, and 200 µm.

### VEGFA expression by immunofluorescence

**Figure 4:**
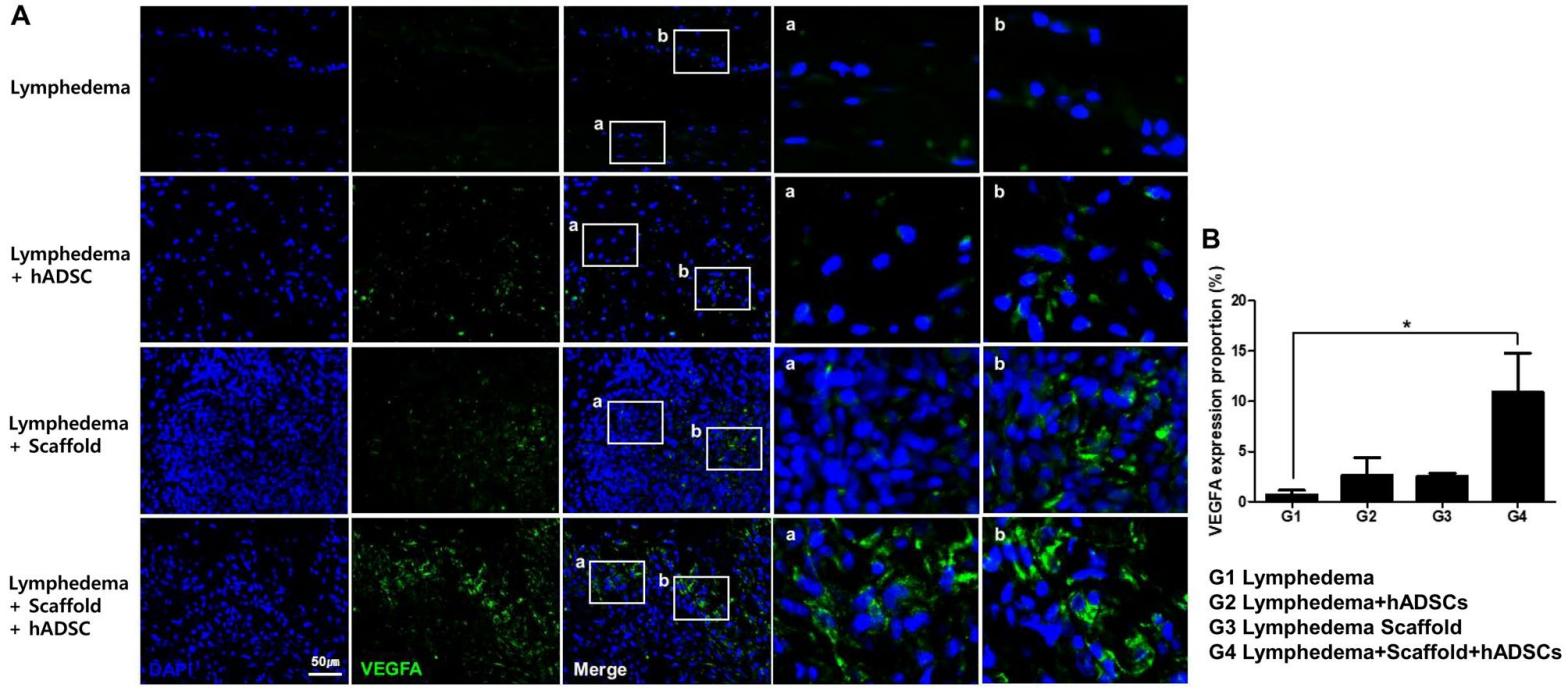
VEGFA expression was assessed by immunofluorescence staining. (**A**) VEGFA was not expressed in lymphedema group. The expression of VEGFA hardly observed in ADSCs injection and Decell LN-scaffold groups. In the Recell LN-scaffold group, VEGFA was significantly expressed in many cells. (**B**) Bar graphs showing that rat in the Recell LN-scaffold group had the greatest number of VEGF-positive cells (*P < 0.05). VEGFA, vascular endothelial growth factor A.

To further confirm the effects of recellularized lymph node-scaffold with hADSCs on angiogenesis, we performed immunofluorescence staining for VEGFA. Compared with the lymphedema and decellularized lymph node-scaffold groups, in which the number of VEGFA-expressing cells was low, the numbers of VEGFA-positive cells were significantly higher in the hADSCs injection and recellularized lymph node-scaffold groups (Fig. 4A). The VEGFA expression was measured manually in five microscopic fields in each stained sample. The VEGFA expression was significantly increased in recellularized lymph node-scaffold group compared with other groups (G1, 0.70 ± 0.43%; G2, 2.63 ± 1.73%; G3, 2.51 ± 0.36%; G4 10.97 ± 3.79%*, *P* < 0.05, Fig. 4B).

### LYVE-1 and human Nuclear expressions by immunofluorescence

**Figure 5:**
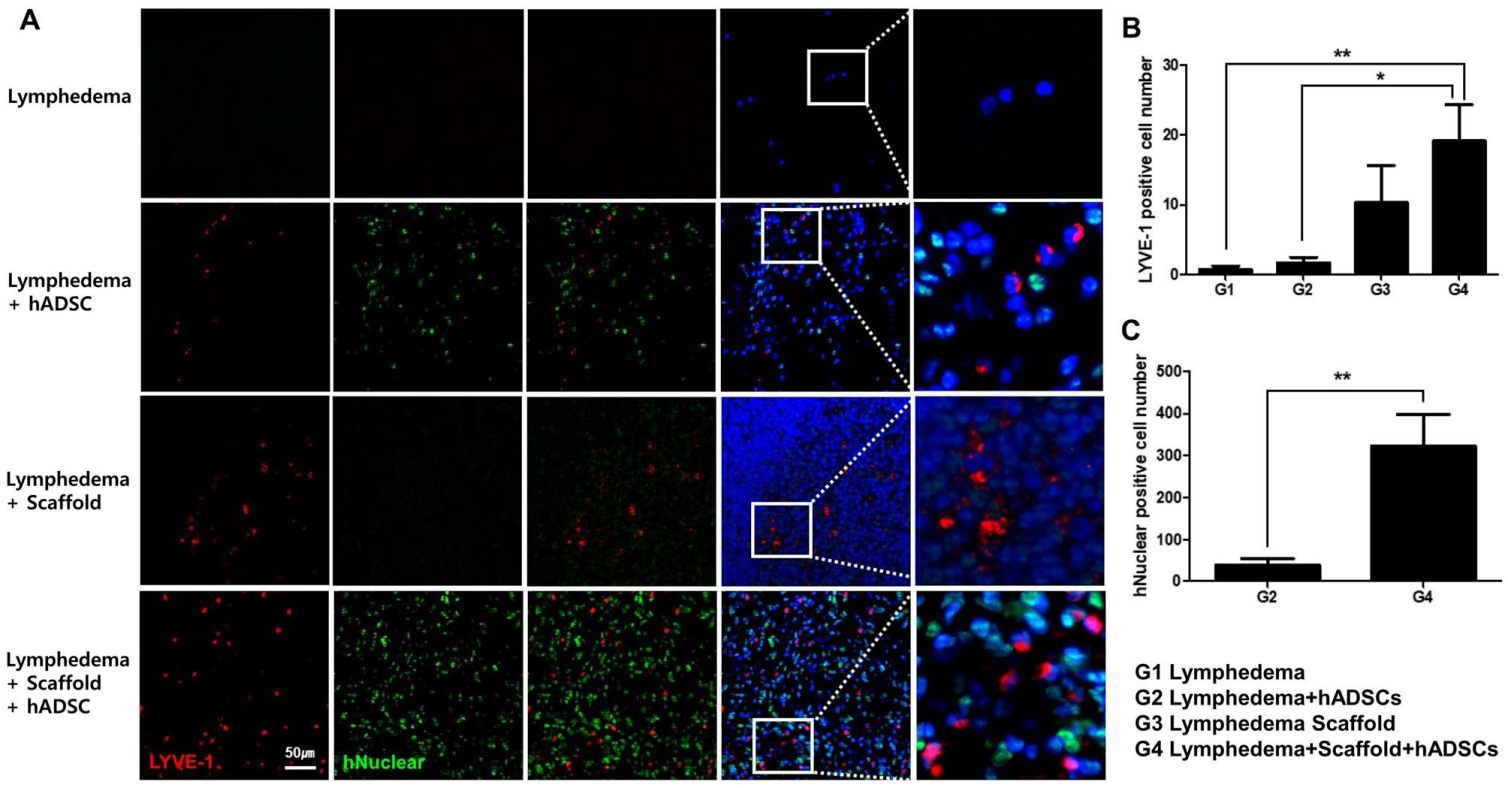
LYVE-1 and human nuclear expressions were assessed by immunofluorescence staining. (**A**) LYVE-1 and hNuclear were not expressed in lymphedema group, and the expression of LYVE-1 was hardly expressed and some positive cells of hNuclear was confirmed in ADSCs treatment group. LYVE-1 was slightly expressed and hNuclear were not expressed in the Decell LN-scaffold group. In the Recell LN-scaffold group, most of the cells were hNuclear positive, and LYVE-1 expression was observed in some of the hNuclear positive cells. (**B,C**) Bar graphs showing that rat in the Recell LN-scaffold group had the greatest number of LYVE-1 and hNuclear-positive cells (*P < 0.05, **P < 0.01). *LYVE-1* lymphatic vessel endothelial hyaluronan receptor 1, *hNuclear* human nuclear.

In order to confirm whether the expression of LYVE-1 associated with lymphadenogenesis was originated from the transplanted hADSCs, it was confirmed by double staining with anti-LYVE-1 and anti-human Nuclear. As there were no hADSCs in lymphedema and decellularized lymph node-scaffold groups, there were no hNuclear-positive cells. In hADSCs treatment group, a relatively large number of hNuclear-positive cells were observed, but LYVE-1 was rarely expressed. In the case of recellularized lymph node-scaffold with hADSCs, a significantly higher number of hNuclear-positive cells were observed than in the hADSCs treatment group, and LYVE-1 were also expresed. Moreover, a large number of cells simultaneously expressing hNuclear and LYVE-1 were observed (Fig. 5A). To quantify LYVE-1 and hNuclear expression, LYVE-1 and hNuclear-positive cells were counted manually in microscopic fields in each stained sample. The expression of LYVE-1 was relatively increased in lymph node scaffold groups. In particular, the expression of LYVE-1 was significantly increased in recellularized lymph node-scaffold group (G1, 0.66 ± 0.49; G2, 1.66 ± 0.76; G3, 10.29 ± 5.36; G4, 19.17 ± 5.20, G1 vs. G4**, G2 vs. G4*, *P < 0.05, **P < 0.01, Fig. 5B). The expression of hNuclear was significantly increased in recellularized lymph node-scaffold group compared with ADSCs injection group (G2, 38.00 ± 19.97 vs. G4, 322.70 ± 75.06**, *P* < 0.01, Fig. 5C).

### LYVE-1and VEGFA protein expression

**Figure 6:**
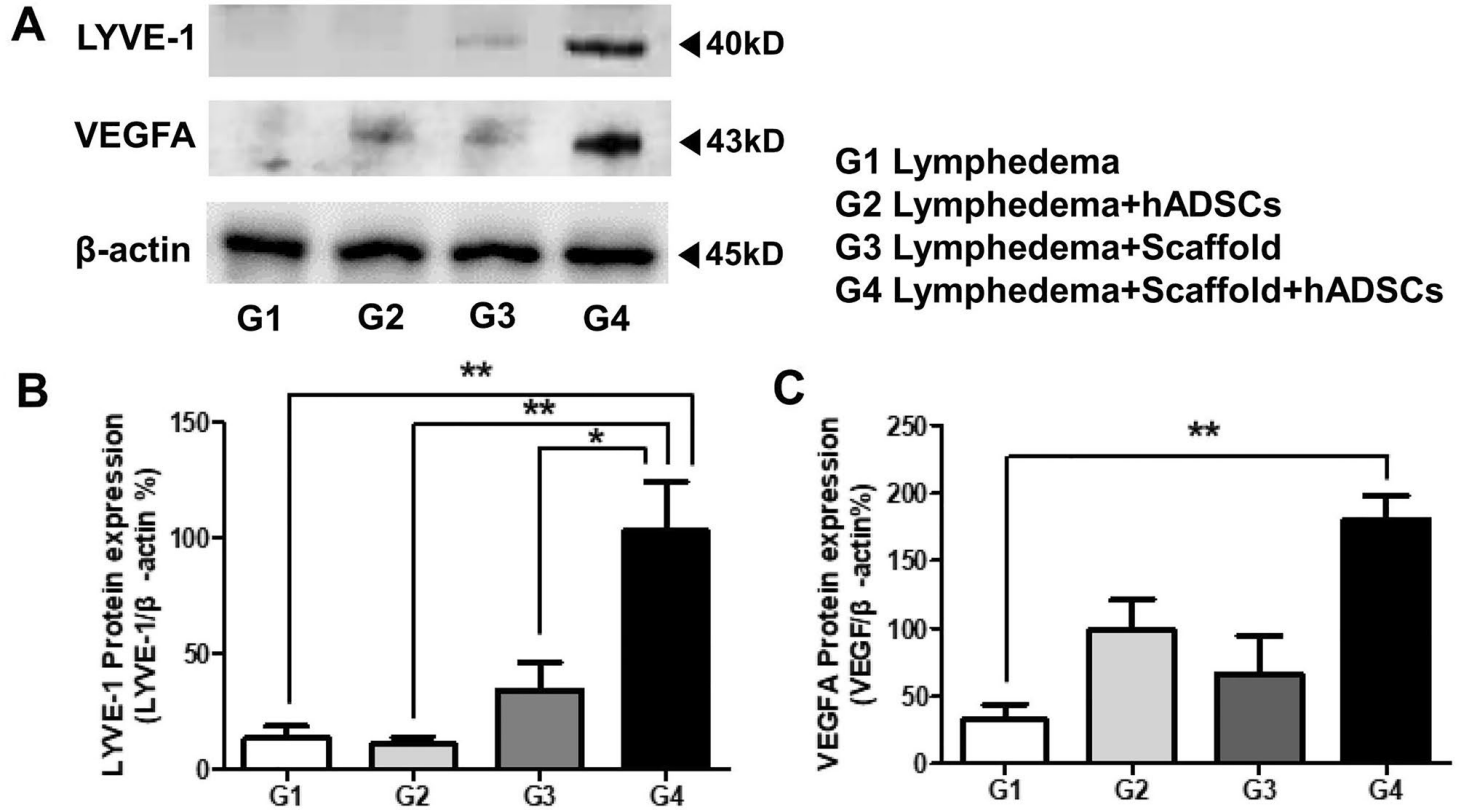
LYVE-1 protein expression after recellularizied lymph node scaffold transplantation. The expression level of the LYVE-1 protein was determined by Western blotting and higher in recellularizied lymph node scaffold group compared with other groups. Bar graphs were analyzed via blot intensities of densitometric scanning. Data are presented as means ± SEM (*P < 0.05, **P < 0.01; n = 5 per group). LYVE-1, Lymphatic vessel endothelial hyaluronan receptor 1. Uncropped blots are included with supplemental data (Supplementary Fig. S2–S4).

The expression level of the LYVE-1 and VEGFA protein associated with lymphadenogenesis was determined by Western blotting. In this analysis, the intensities of the blots were determined via densitometric scanning, and relative densities were expressed as ratios relative to the endogenous control value. The results of western blotting revealed that the expression of LYVE-1 protein significantly increased in recellularized lymph node-scaffold group compared with others (G1, 14.09 ± 4.88**; G2, 11.36 ± 2.89**; G3, 34.69 ± 11.99* vs G4, 103.20 ± 21.40, *P < 0.05, **P < 0.01, Fig. 6A,B). The expression pattern of VEGFA in Western blot was similar to that of tissue staining, and it was significantly increased in the recellularized lymph node-scaffold group (G1, 32.19 ± 11.65; G2, 98.32 ± 22.77; G3, 66.25 ± 28.43; G4, 180.01 ± 18.02**, **P < 0.01, Fig. 6A,C). Uncropped blots are included with supplemental data (Supplementary Fig. S2–S4).

## Discussion

The lymphatic circulation plays an important role in transporting lymph fluid, including leukocytes, proteins, and lipids, from the interstitial space to the central venous circulation^16^. Lymphatic vessels contract rhythmically, transporting lymph and inhibiting reflux by valves. When there is a problem with the lymphatic circulation, fluid accumulates in the interstitial space^17^. Recently, the incidence of lymphedema is increasing due to surgery and radiotherapy related to malignant neoplasms^11,16^. Most of the standard treatment for lymphedema is physiotherapy such as lymphatic drainage and compression bandage. Recently, although reconstructive microsurgery, lymphatic transplantation, cell therapy, and scaffold transplantation have been proposed, many problems remain to be solved for clinical^12–14,18^.

We fabricated a three-dimensionally structured biocompatible scaffold by decellularized lymph node, and investigated the lymph node reconstruction effect by recellularizing hADSCs to maximize the therapeutic effect. Axillary lymph node was decellularized with 0.075% SDS, and it was confirmed that most of the cellular components were removed from hematoxylin and eosin staining. In addition, it was confirmed that the three-dimensional structure was well preserved in masson's trichrome staining.

Previous researchers studied the effects and benefits of the recellularized scaffolds^19^. A variety of cells was used for recellularization. Lin et al. recellularized BM-MSCs in decellularized lymph node and cultured for 3 days. It has been reported that BM-MSCs were well grafted inside decellularized lymph node and induce a mature and robust immune response when stimulated with antigen^10^. We hypothesized that recellularization of decellularized lymph node with hADSCs would promote lymph node regeneration with the ability of stem cells. In this study, we tried to overcome the limitation of cell loss by injecting hADSCs into decellularized lymph node scaffolds, culturing recellularized lymph nodes in an incubator for 3 days, and then transplanting them into animals. On the 3rd and 7th days of recellularization, the presence or absence of ADSCs in the recellularized lymph node scaffold was confirmed by staining. Direct injection of hADSCs into the lymph node or around the lymph node has limitations in low cell survival and engraftment due to cell loss. More hADSCs were observed on day 7 than on day 3 in the recellularized lymph node scaffold, indicating that hADSCs were stably engrafted and proliferated on the recellularized lymph node scaffold. We transplanted the recellularized lymph node scaffolds injected with PKH26-labeled hADSCs into lymphedema animals to clearly confirm the cell loss inhibitory effect of the recellularized lymph node scaffolds and followed the ADSCs on postoperative day 7 and 14. In the hADSCs group, few hADSCs were observed. However, many hADSCs were observed in the recellularized lymph node-scaffold group. Therefore, it can be said that the strategy of recellularization on the scaffold is more effective than the direct transplantation of stem cells.

In histopathological observation, there were no signs of lymph node regeneration in the control and hADSCs groups. However, after transplantation of decellularized or recellularized lymph node scaffolds, lymph node shape was well maintained and lymph node reconstruction was in progress. A large number of hADSCs were observed in the hADSCs injection group, but the lymph node structure could not be regenerated. However, collagen deposition and vascular structures were observed around the hADSC injection site, and structures such as lymph node capsules were identified. Previous investigators have reported that hADSCs injection improved lymphedema in an animal model of lymphedema^7,20^. In our results, the effect of hADSCs injection was not significant. Previous studies focused on lymphangiogenesis, and we aimed to regenerate the lymph nodes, a more difficult work for tissue regeneration. This is not to say that hADSCs injections are ineffective. Furthermore, this is probably because there is a lot of loss of stem cells by direct injection, and the only stem cells are insufficient to reconstruct the removed lymph nodes. We demonstrated the effect of a recellularized lymph node scaffold to minimize the loss of stem cells and maximize lymph node regeneration by using a scaffold that can regenerate the lymph node. As in this study, when reconstructing some tissues, it is more effective to treat the scaffold and stem cells together than to treat the stem cells alone. The decellularized lymph node-scaffold transplant group maintained the lymph node structure, but it was different from the normal lymph node due to unclear capsule boundaries and agglomerate of lymphocytes and fibroblasts. It can be confirmed that this is a transplanted scaffold, and decellularized lymph node-scaffold is more efficient in lymph node regeneration than stem cell injection. Decellularized scaffolds have a three-dimensional structure containing macromolecules such as collagen, elastin, and fibronectin, and are known to be advantageous for repair of damaged organs and regeneration of endogenous tissues^21^. In the decellularized lymph node-scaffold, various ECM and 3D structures seem to play a role in lymph node regeneration. In the recellularized lymph node-scaffold group, the lymph node regeneration was the most ideal pattern. Recellularized lymph node-scaffold was histologically similar to normal lymph nodes compared to other groups, and showed a more complete lymphatic regeneration effect. The hADSCs were observed inside the scaffold, confirming the recellularized lymph node-scaffold we transplanted. According to another researcher’s study, recellularized ECM scaffolds can induce the site-specific differentiation of stem cells and coordinate with cells and tissues^21^. It seems that our results were also able to maximize the advantages of the stem cell and scaffold combination.

VEGF induces strong angiogenesis and is secreted from hADSCs along with other growth factors. Angiogenesis plays an important role in promoting tissue regeneration^22^. In this study, the VEGFA expression pattern was investigated to determine whether hADSCs or scaffolds promote lymph node regeneration. In the decellularized lymph node-scaffold group, VEGFA expression could be confirmed, but it was insufficient. VEGFA was highly expressed in hADSCs treatment groups. In particular, VEGFA was clearly expressed in the recellularized lymph node-scaffold group, which seems to promote lymph node regeneration by supplying sufficient blood into the lymph nodes. Cursiefen et al. reported that LECs can express VEGFR2 and that VEGFA supports lymphatic survival^23^. Nagy et al. reported that VEGFA overexpression led to the formation of large lymphatic vessels^24^. In this study, the expression of VEGFA protein was confirmed using Western blot. The expression pattern of VEGFA in Western blot was similar to that of tissue staining, and it was significantly increased in the recellularized lymph node-scaffold group. Although we did not investigate lymphangiogenesis in our results, it is believed that VEGFA expression by hADSCs contributed to not only angiogenesis but also lymphangiogenesis.

To confirm lymph node reconstruction, the staining using the LYVE-1 marker was performed. LYVE-1 is a selective marker of lymphatic endothelium and binds hyaluronic acid on the luminal face of the lymphatic vessel wall^25^. It is a strong specific lymphatic vessel marker that is not normally found in blood vessels^26^. There was no expression of LYVE-1 in the control group, and LYVE-1 was slightly expressed in the hADSCs and decellularized lymph node-scaffolds groups. LYVE-1 was significantly expressed in the recellularized lymph node-scaffold group compared to other groups. According to Zhou et al., the hADSCs promote VEGFR3-mediated lymphangiogenesis and proliferation of lymph endothelial cell through the METTL3 pathway^27^. So, we performed the double-stained with the hNuclear marker to confirm the relationship between hADSCs treatment and LYVE-1 expression. A large number of hNuclear positive cells were significantly observed in the recellularized lymph node-scaffold compared with hADSCs injection group. Similarly, it was confirmed by western blot that the LYVE-1 expression was significantly increased in recellularized lymph node-scaffold group. Decellularized lymph node-scaffolds partially induce LYVE-1 expression, but hADSCs seem to contribute more effectively to LYVE-1 expression. Moreover, the synergistic effect of hADSC and scaffold in recellularized lymph node-scaffold significantly increased the expression of LYVE-1, which promotes lymph node regeneration. This suggests that recellularized lymph node-scaffold recellularized with hADSC promotes VEGFA expression and VEGFR3-mediated lymphangiogenesis, and contributes to lymph node regeneration through activation of LYVE-1 expression. We confirmed the possibility of lymph node regeneration using recellularized lymph node-scaffolds. The use of lymph node scaffolds and stem cells to treat lymphedema will be a new strategy for lymph node regeneration.

A limitation of this study is that we only checked the high expression of VEGFA and LYVE-1, not the functional aspect of these recellularized scaffolds. According to previous reports, LYVE-1 is mainly stained in lymphatic vessels and is observed in the form of tubes. However, our target was the area of the removed lymph node or lymph node scaffold; therefore, we could only confirm the expression level rather than the tube-like shape. Creating actual lymphedema in the rat legs is challenging. To produce clinically relevant lymphedema in rat legs, severe circulation disturbances must be sustained over a long time. In addition, the leg circumference was very small, and the leg circumference measurement value was significantly different based on leg posture. Moreover, the authors thought that evaluating the lymphatic flow by injecting dye was the best way to measure the outcomes and tried implementing it. However, we found that the surrounding tissues were also entirely contaminated with the dyes, and in this case, performing a histopathological assessment and an immunofluorescence analysis was impossible.

Regenerative medicine research using stem cells and scaffolds is being widely studied as an important treatment, and some are being applied to clinical trials or patients. We produced the hADSCs-based recellularized lymph node-scaffolds and investigated the effects of lymph node regeneration in lymphedema rat models. Recellularized lymph node-scaffold had a much better therapeutic effect than stem cells or decellularized scaffold alone, and induced stable stable lymphangiogenesis in a short time. Although further mechanistic studies are essential, our results suggest a new strategy for the treatment of lymphedema patients.

## Materials and methods

### Animals

Sprague Dawley male rat (SD, 7 weeks old, 220–250 g) were purchased from ORIENT BIO (Seongnam, Republic of Korea) and maintained according to the Association for Assessment and Accreditation of Laboratory Animal Care International system. All animal experiments confirmed to the International Guide for the Care and Use of Laboratory Animals and were approved by the Institutional Animal Care and Use Committee of Seoul National University Bundang Hospital (IACUC No. BA-1908-277-061-01). This study was reported in accordance with ARRIVE guidelines.

### hADSC isolation and characterization

The hADSCs were isolated from three patients who underwent abdominal surgery. This study was approved by Seoul National University Bundang Hospital Institutional Review Board and Ethics Committee and conducted in accordance with the guidelines of the 1975 Declaration of Helsinki (IRB No. B-1603/338-309). Written informed consent was obtained from all subjects. Briefly, adipose tissue was washed with phosphate-buffered saline (PBS) and cut into smaller pieces. Enzymatic digestion was performed using 0.075% collagenase type I (Sigma-Aldrich, St. Louis, MO, USA) in a humidified 5% CO_2_ incubator for 1 h at 37 °C. After neutralization, samples were centrifuged, and supernatants were passed through a 100 µm cell strainer (BD Bioscience, Bedford, MA, USA). The cells were transferred into cell culture flasks with Dulbecco’s modified Eagle’s medium (Welgene, Gyeongsan, Republic of Korea) supplemented with 10% fetal bovine serum (Gibco, Carlsbad, CA, USA), 100 U/mL penicillin, and 100 μg/mL streptomycin (Lonza, Walkersville, MD, USA); cells were maintained at 37 °C in a humidified 5% CO_2_ incubator. The hADSCs were used between 4 and 6 passages for FACS and animal experiments. hADSCs were characterized by flow cytometry for the cell surface markers CD31, CD34, CD44, CD45, CD90, and HLA-DR (BD Biosciences Pharmingen, San Jose, CA, USA, Supplementary Fig. S1).

### Lymph node decellularization and recellularization

Axillary lymph node were harvested from SD rats (n = 5) for decellularization. The long diameter of axillary lymph nodes for decellularization was 4–5 mm and the short diameter was 2–2.5 mm. The lymph nodes were washed 3 times in PBS to remove blood and debris. The decellularization protocols followed the modified method reported by Cuzzone et al.^9^. The decellularized lymph nodes were performed in a solution of 0.075% sodium dodecyl sulfate (SDS) / PBS for 15 h at 37 °C in an orbital shaker at 150 rpm and washed with PBS three times. The recellularization of lymph nodes performed that PKH26-labeled hADSCs (1 × 10^6^/50 µL) were injected in the decellularized lymph node. An insulin syringe (30G needle) was used for the hADSC injection. Without specifying the entry point, the solution was injected three or four times into the cortex. The recellularized lymph nodes were not immediately implanted, but were incubated in a 5% CO_2_ incubator at 37 °C to recellularize for 3 days and then were implanted. The recellularized lymph nodes were fixed in 10% formalin and embedded in Scigen O.C.T. Compound Cryostat Embedding Medium (Scigen Scientific, Gardena, CA, USA). The tissues were routinely processed and cut into sections 10–15 μm thick using LEICA CM 1860 Cryostat Microtome (Leica, Buffalo Grove, IL, USA).

### Lymphedema animal models

**Figure 7:**
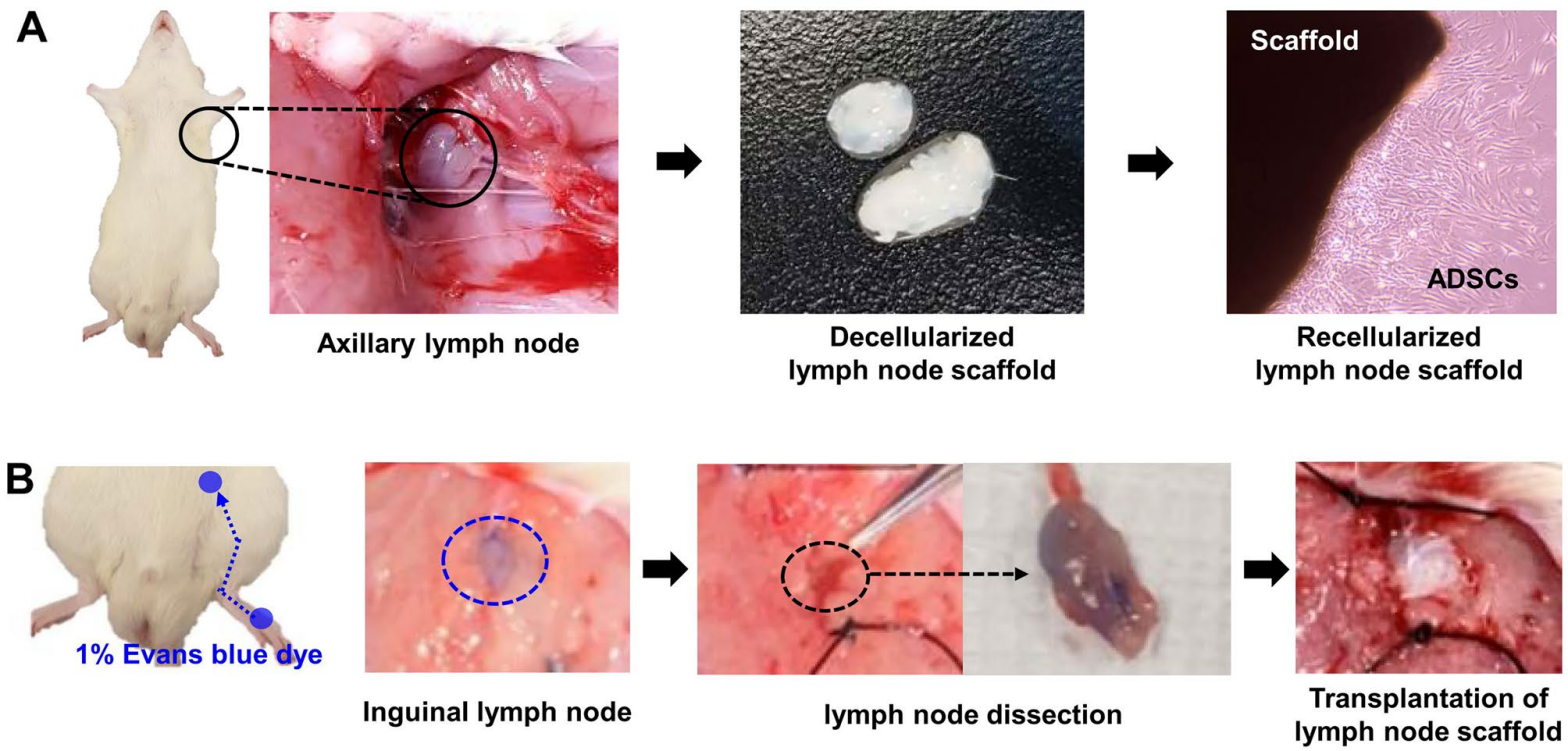
The process of recellularizied lymph node scaffold transplantation in lymphedema rats. (**A**) Axillary lymph nodes were harvested from SD rat and decellularization was performed. The scaffolds were recellularized by ADSCs. (**B**) 1% Evans blue was injected into the dorsum of paw and inguinal lymph nodes were removed. The recellularized lymph node scaffold was transplanted at the site of lymph node removal.

Forty SD rats were equally divided into four groups: lymphedema as control group (G1), hADSC group (G2), decellularized lymph node-scaffold group (G3), and recellularized lymph node-scaffold group (G4). The lymphedema animal model followed the method reported by Yang et al.^28^. SD rats were under inhalation anesthesia using 2% isoflurane. In order to facilitate the identification of the location of the lymph nodes, 10% Evans blue dye (Glentham Life Sciences, Corsham, London, UK) was injected subcutaneously into both instep of the rat. After both femoral incisions, inguinal lymph nodes are identified, and lymph nodes in both femurs are removed using a microscopy. Depending on the group, ADSC injection or decellularized lymph node-scaffold or recellularized lymph node-scaffold is implanted and the skin was sutured (Fig. 7). To track the transplanted hADSCs, we labeled them with PKH26 red fluorescent dye according to the manufacturer’s instructions. Briefly, hADSCs (1 × 10^7^/mL) were harvested and resuspended in 1 mL of diluent C solution. Then, 4 µL PKH26 dye was added followed by incubation for 5 min. Fetal Bovine Serum (1 mL) was added to quench for 2 min, followed by washing with PBS. The proportion of PKH26-positive cells was measured using Image J software (version 1.53K; National Institutes of Health, Bethesda, MD, USA) in five microscopic fields in each stained sample.

### Histopathological assessment

Tissues were fixed in 10% formalin and embedded in paraffin. The tissues were routinely processed and cut into 4–5 μm-thickness sections. The sections were deparaffinized in xylene at room temperature and stained with hematoxylin and eosin (Cancer Diagnostics Inc., Durham, NC, USA) according to the manufacturer’s instructions. Masson’s trichrome staining (BBC Biochemical, Mount Vernon, WA, USA) was performed following the manufacturer’s protocol. Briefly, deparaffinized sections were fixed in Bouin’s solution for 1 h at 56 °C and stained with ClearView Iron Hematoxylin working solution for 10 min. Subsequently, tissues were stained with Biebrich scarlet-acid fuchsin solution (2 min), phosphomolybdic–phosphotungstic acid solution (10 min), aniline blue solution (3 min), and 1% acetic acid solution (2 min). ECM, collagen, and other connective tissue elements were stained in blue and smooth muscles were stained in red. Tissue sections were imaged (400× magnification) using slide scanner (3DHISTECH Ltd, Budapest, Hungary).

### Immunofluorescence analysis

Tissue sections were deparaffinized in xylene and rehydrated in a graded ethanol series. After heat-induced epitope retrieval in citrate buffer, pH 6.0 (Scytek Laboratories, Inc. West Logan, UT, USA) for 40 min, sections were incubated with 3% bovine serum albumin blocking reagent for 10 min at room temperature. Subsequently, tissue sections were incubated with anti-vascular endothelial growth factor A (VEGFA) (Abcam, Cambridge, UK), anti-Lymphatic vessel endothelial hyaluronan receptor 1 (LYVE-1, NOVUS, Briarwood, CO, USA), and anti-human Nuclear (Biorbyt, Cambridge, UK) primary antibodies (1:100) for 90 min at room temperature, followed by incubation with Alexa Fluor 488 (Biolegend, San Diego, CA, USA) and Alexa Fluor 594 (Biolegend, San Diego, CA, USA) secondary antibody (1:500). The sections were mounted with DAPI-containing mounting medium (Vector Laboratories Inc., Burlingame, CA, USA) and observed under an inverted microscope (Zeiss MicroImaging GmbH, Jena, Germany). The proportion of VEGFA-positive cells was measured using Image J software (version 1.53K; National Institutes of Health, Bethesda, MD, USA) in five microscopic fields in each stained sample. LYVE-1 and hNuclear-positive cells were counted manually in five microscopic fields in each stained sample, and the mean value was used for statistical analyses.

### Western blotting

The tissues were homogenized using PROPREP lysis buffer (Intron, Seoul, Republic of Korea), and the concentration of cellular protein was determined using the Bio-Rad assay reagent (Bio-Rad, Hercules, CA, USA). Briefly, samples with equal concentrations of protein were mixed with 4× sample buffer (GenDEPOT Inc., Barker, TX, USA), heated at 95 °C for 10 min, and separated by 10% sodium dodecyl sulfate–polyacrylamide gel electrophoresis (SDS-PAGE). Proteins were then transferred onto Polyvinylidene fluoride membrane (Merck Millipore, Darmstadt, Germany) in Tris–glycine transfer buffer (Invitrogen, Carlsbad, CA, USA). The membranes were blocked for 1 h at room temperature with 5% Bovine Serum Albumin (BSA) (Millipore, Kankakee, IL, USA). The membranes were incubated at 4 °C overnight with anti-LYVE-1 (NOVUS, Briarwood, CO, USA) and anti-β-actin (Santa Cruz Biotechnology, Santa Cruz, CA, USA) primary antibodies, followed by incubation with horseradish peroxidase-conjugated anti-mouse IgG (Cell Signaling Technology, Danvers, MA, USA) or anti-rabbit secondary antibody (Santa Cruz Biotechnology, Santa Cruz, CA, USA) secondary antibodies as appropriate for 1 h at room temperature. The membranes were washed and then incubated using a West-Q Chemiluminescent Substrate Plus kit (GenDEPOT Inc., Barker, TX, USA). The intensities of the protein bands were determined using Multi Gauge software (version 3.0; Fuji Photo Film, Tokyo, Japan); relative densities were expressed as ratios of control values. All reactions were performed in triplicate.

### Statistical analysis

Quantitative data were expressed as mean ± standard deviation. Differences between groups were evaluated by one-way analysis of variance (ANOVA) followed by Dunn’s multiple comparison post-hoc tests and student’s t-test. *P*-values < 0.05 were considered statistically significant. All statistical analyses were performed using PRISM v.5.01 (GraphPad Software, Inc., La Jolla, CA, USA) (Supplementary Information).

### Institutional review board statement

All experimental procedures were approved by the Institutional Animal Care and Use Committee of Seoul National University Bundang Hospital (IACUC No. BA-1908-277-061-01). This study was approved by Seoul National University Bundang Hospital Institutional Review Board and Ethics Committee and conducted in accordance with the guidelines of the 1975 Declaration of Helsinki (IRB No. B-1603/338-309).

## Supplementary Information

Supplementary Figures.

## Data Availability

The datasets used and analysed during the current study available from the corresponding author on reasonable request.
