# Supplementary material for: Recellularized lymph node scaffolds with human adipose-derived stem cells enhance lymph node regeneration to improve lymphedema
Source: Sci Rep. 2023 Apr 3;13:5397. doi: 10.1038/s41598-023-32473-z (PMC10070624; doi:10.1038/s41598-023-32473-z)
Supplement: Supplementary file 1 — Supplementary Figures. [file 41598_2023_32473_MOESM1_ESM.docx]

**<Supplementary Information>**

Recellularized lymph node scaffolds with human adipose-derived stem cells enhance lymph node regeneration to improve lymphedema

**Hyo Jin Kang^1^, Soo Young Moon^1^, Baek-Kyu Kim^2^, Yujin Myung^2^, Ju-Hee Lee^3^,**

**and Jae Hoon Jeong^2,^***

^1^ Department of Biomedical Laboratory Science, Honam University, Gwangju, 62399, Republic of Korea

^2^ Department of Plastic and Reconstructive Surgery, Seoul National University Bundang Hospital, Seongnam, Gyeonggi-do, 13620, Republic of Korea

^3^ College of Korean Medicine, Dongguk University, Goyang, Gyeonggi-do, 10326, Republic of Korea

***Corresponding author:**

Jae Hoon Jeong, MD, Ph D.

Seoul National University Bundang Hospital

82, Gumi-ro 173 Beon-gil, Bundang-gu

Seongnam-si Gyeonggi-do 13620, Republic of Korea

psdrj2h@gmail.com


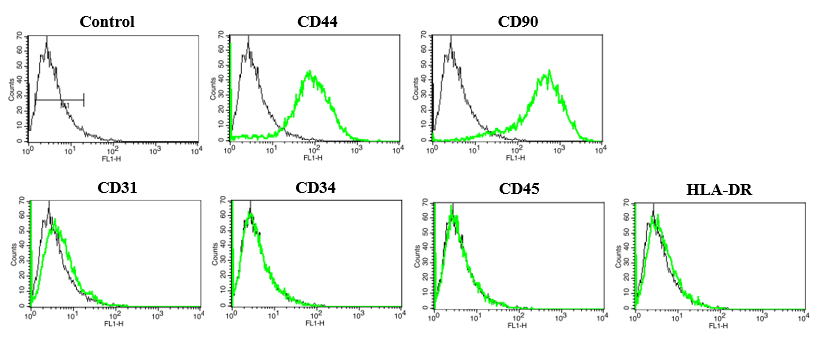


**Figure S1. hADSCs characterization.** The hADSCs were identified based on cell surface markers. Positive markers of primary cultured hADSCs included CD44 and CD90; the negative markers were CD31, CD34, CD45, and HLA-DR.


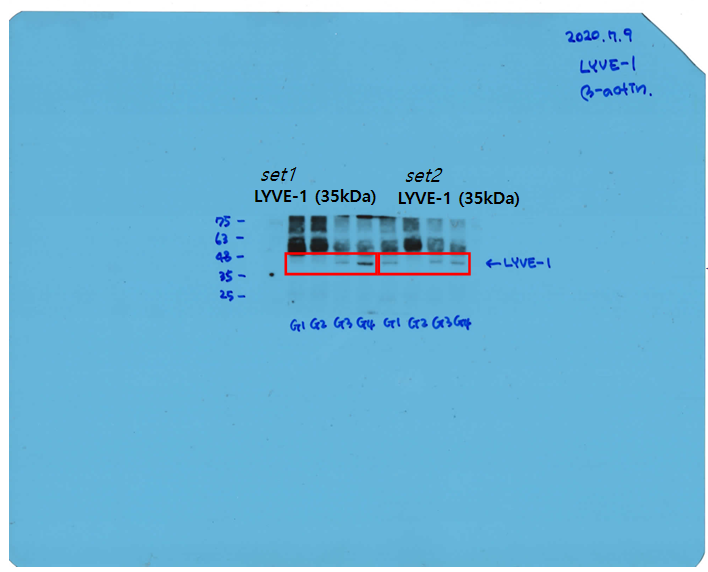


# Figure S2. The Western blot of LYVE-1 in the four group. The original uncropped western blot images of LYVE-1. LYVE-1, Lymphatic vessel endothelial hyaluronan receptor 1.


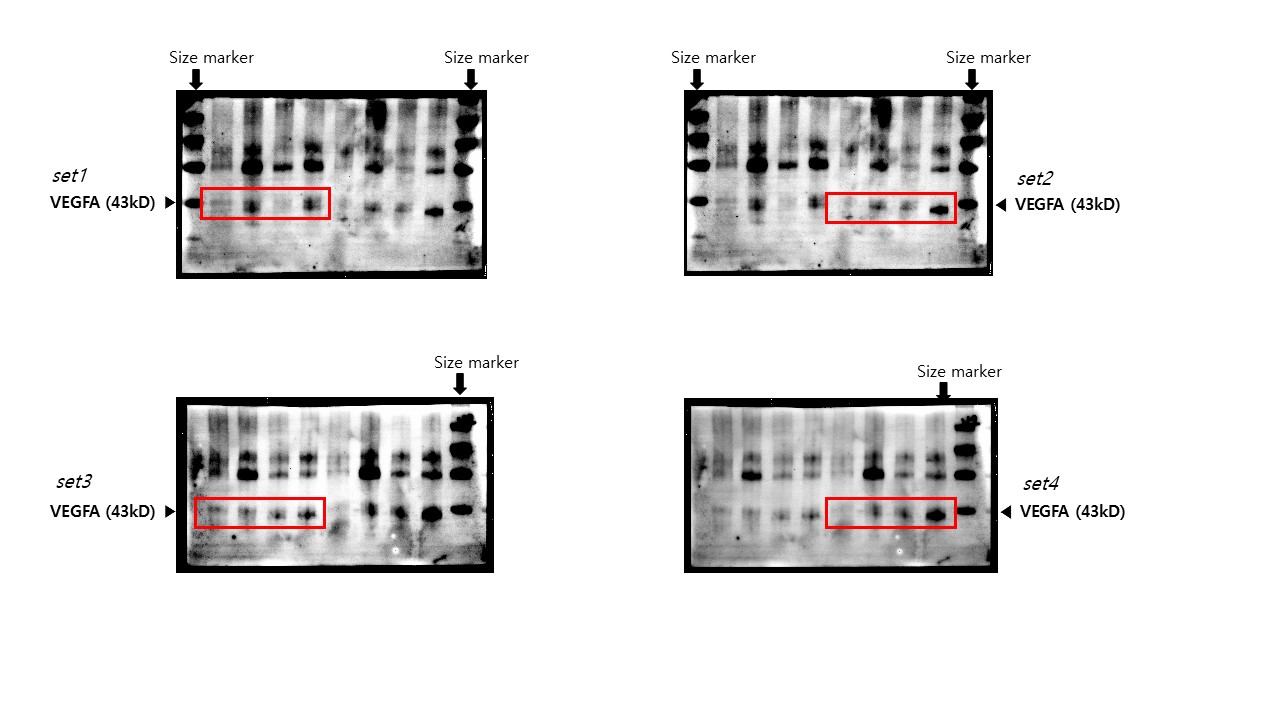


# Figure S3. The Western blot of VEGFA in the four group. The original uncropped western blot images of VEGFA. VEGFA, vascular endothelial growth factor A.


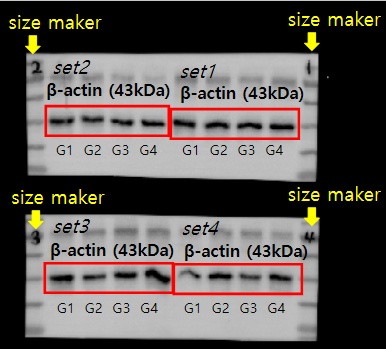


# Figure S4. The Western blot of β–acting in the four group. The original, uncropped western blot images of β–acting.
